# Subclinical psoriatic arthritis and disease interception—where are we in 2024?

**DOI:** 10.1093/rheumatology/keae399

**Published:** 2024-08-16

**Authors:** Clementina López-Medina, Dennis McGonagle, Laure Gossec

**Affiliations:** Medical and Surgical Sciences Department, University of Cordoba, Cordoba, Spain; Department of Rheumatology, Reina Sofia University Hospital, Maimonides Institute for Biomedical Research of Cordoba, Cordoba, Spain; Institut Pierre Louis d'Epidémiologie et de Santé Publique, Sorbonne Université, INSERM, Paris, France; NIHR Leeds Biomedical Research Centre; Leeds Institute of Rheumatic and Musculoskeletal Medicine, University of Leeds, Leeds, UK; Institut Pierre Louis d'Epidémiologie et de Santé Publique, Sorbonne Université, INSERM, Paris, France; Rheumatology Unit, Pitié-Salpêtrière University Hospital, AP-HP, Paris, France

**Keywords:** spondyloarthritis, spondylarthropathies, psoriatic arthritis, skin, epidemiology, DMARDs

## Abstract

Psoriatic arthritis (PsA) is a chronic rheumatic disease that usually appears in patients with skin psoriasis, making it a model for detection of joint disease in the pre-clinical phases in a setting where therapy for cutaneous disease may ameliorate or prevent arthritis development. Such PsA prevention appears credible due to the increasingly recognized closely shared immunopathology between the skin and joints, especially the entheses. Recently, several initiatives have explored the concept of pre-clinical PsA, and nomenclatures have been developed with the recent EULAR nomenclature proposing a simplified three stages from psoriasis to clinical PsA development, namely at risk of PsA, subclinical PsA and early PsA. A better comprehension of early PsA and the identification of individuals predisposed to its development could enable interventions to ‘prevent’ the appearance of PsA. Several recent retrospective observational studies have demonstrated disease interception feasibility, i.e. treatment of people with psoriasis may prevent the appearance of PsA, in particular using biologic disease-modifying drugs. However, further data are urgently required due to unexpected findings in some studies where TNF inhibition for psoriasis does not reduce the rate of PsA development. In this review we address the current challenges in early PsA, including comparisons of pre-PsA nomenclature sets, its risk factors and the potential for disease interception.

## Graphical Abstract

**Figure:**
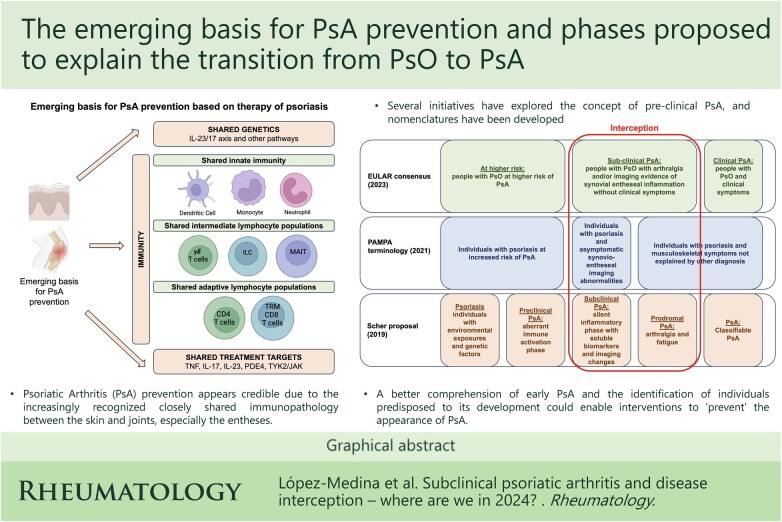

Rheumatology key messages

- Several initiatives have explored the concept of pre-clinical PsA, with slight differences in definitions.
- EULAR suggested a simplified concept with three stages: ‘at risk’, subclinical and early PsA.
- The identification of individuals ‘at risk’ could enable interventions to ‘prevent’ the appearance of PsA.

## Introduction

Psoriatic arthritis (PsA) is a chronic rheumatic disease usually associating skin psoriasis (PsO) and arthritis [1]. The heterogeneity in the clinical picture may pose challenges for PsA diagnosis, which currently rests on classification criteria rather than diagnostic criteria. PsA usually starts in patients previously presenting with skin PsO. Indeed, over their lifetime, ∼30% of individuals with PsO will develop PsA [2]. The incidence of PsA among individuals with PsO is relatively stable over time and is estimated between 0.27 and 2.7 per 100 person-years [3]. The incidence is highest among patients aged 30–60 and is generally equally distributed between men and women [4]. Because PsO is a major risk factor for PsA, this disease can be considered a model for detection in pre-clinical phases. The interest in preventing autoimmune disease goes back decades, but what is unique about PsA prevention is the requirement for skin directed psoriasis therapy, meaning that patients who are otherwise completely healthy are not receiving biologic therapy for an asymptomatic disease.

Recently, several initiatives have explored the pre-clinical phase of PsA. The interconnected inflammatory pathways between PsO and PsA, as well as the recognition of pre-clinical phases, suggest a continuum in PsA development. Understanding this pathophysiology and identifying PsO patients at risk of progressing to PsA could facilitate targeted drugs interventions aimed at intercepting the arthritis. Several studies have explored the potential of disease interception in patients with PsO by treatment with disease-modifying drugs. However, their retrospective design prevents definitive conclusions. In this review we address the current challenges in early PsA, including comparisons of pre-PsA nomenclature sets, its risk factors, and the potential for disease interception.

## Early identification of PsA

Early PsA identification may be challenging for clinicians due to heterogeneous manifestations, the irregular presence or absence of elevated acute phase-reactants, the absence of autoantibodies or other robust serum biomarkers and the lack of clinical synovitis in certain cases. Early diagnosis is important in the context of the management of PsA. The concept of a ‘window of opportunity’ has been well-established in rheumatoid arthritis (RA) but has less supportive data in PsA, though current guidelines recommend treating as early as possible [5–7]. This is based on data indicating that a delay of >6 months from onset of symptoms to the first visit contributes to the development of peripheral joint erosions and poorer long-term physical function [8].

Historically, the Moll and Wright criteria (inflammatory arthritis in presence of PsO and negative test for rheumatoid factor) were used to diagnose PsA, though their performance have never been confirmed [9]. Currently, the Classification Criteria for Psoriatic Arthritis (CASPAR) are widely used, mostly in research and in clinical trials [10]. However, it should be noted that these are classification criteria that should be applied after the clinical diagnosis made by the physician. These criteria necessitate the presence of articular, axial or enthesis inflammation as entry criterion, with a minimum of three points derived from the following features: current, previous or family history of PsO; psoriatic nail dystrophy; negative rheumatoid factor; dactylitis; and radiographic evidence of juxta-articular new bone formation [10].

While the CASPAR criteria offer strengths, they also come with limitations. One advantage is their ability to classify patients as PsA in those without skin PsO, incorporating elements such as positive family history of PsO and dactylitis to enhance sensitivity [11]. Moreover, their sensitivity and specificity in established PsA allows for their application as entry criteria in clinical trials and research. However, one weakness lies in their low sensitivity for detecting early PsA, as patients with short disease duration may not exhibit all the typical features. Additionally, there is no clear definition for spine inflammation in the CASPAR criteria. This is in keeping with the current lack of consensus on the definition of axial involvement in PsA [12]. The limitations around spinal diagnosis are mitigated against by the low incidence of isolated axial PsA involvement as a presenting feature of PsA [13]. For these reasons, the CASPAR criteria are not recommended as a diagnostic or screening tool for early arthritis.

Rheumatologists recognize that many inflammatory disorders including RA, connective tissue disease (CTD)-related arthritis and PsA may be accompanied by preceding arthralgia ranging from ‘inflammatory arthralgia’ (defined as joint pain in the early morning together with morning stiffness and with improvement during the day) to non-specific joint pain [14]. Since PsA affects up to a third of people with PsO and their initial symptoms are usually arthralgia, several screening tools and questionnaires have been developed for dermatologists, who are at the forefront of screening for PsA. However, these tools are not widely used in practice. Some examples are the Psoriatic Arthritis Screening and Evaluation (PASE) questionnaire [15], the Psoriasis Epidemiology Screening Tool (PEST) [16], the Toronto Psoriatic Arthritis Screening (ToPAS) [17], the Early Arthritis for Psoriatic patients (EARP) screening questionnaire [18] and the Psoriatic Arthritis UnclutteRed Evaluation (PURE-4) [19]. A study evaluating the performance of these tools found a high prevalence of undiagnosed PsA in patients with PsO (∼29%) [20]. However, the performance of these questionnaires in identifying patients with non-polyarticular presentations of PsA was poor [20].

## Pre-clinical PsA phase: recent advances in definitions and nomenclature

**Figure 1.**
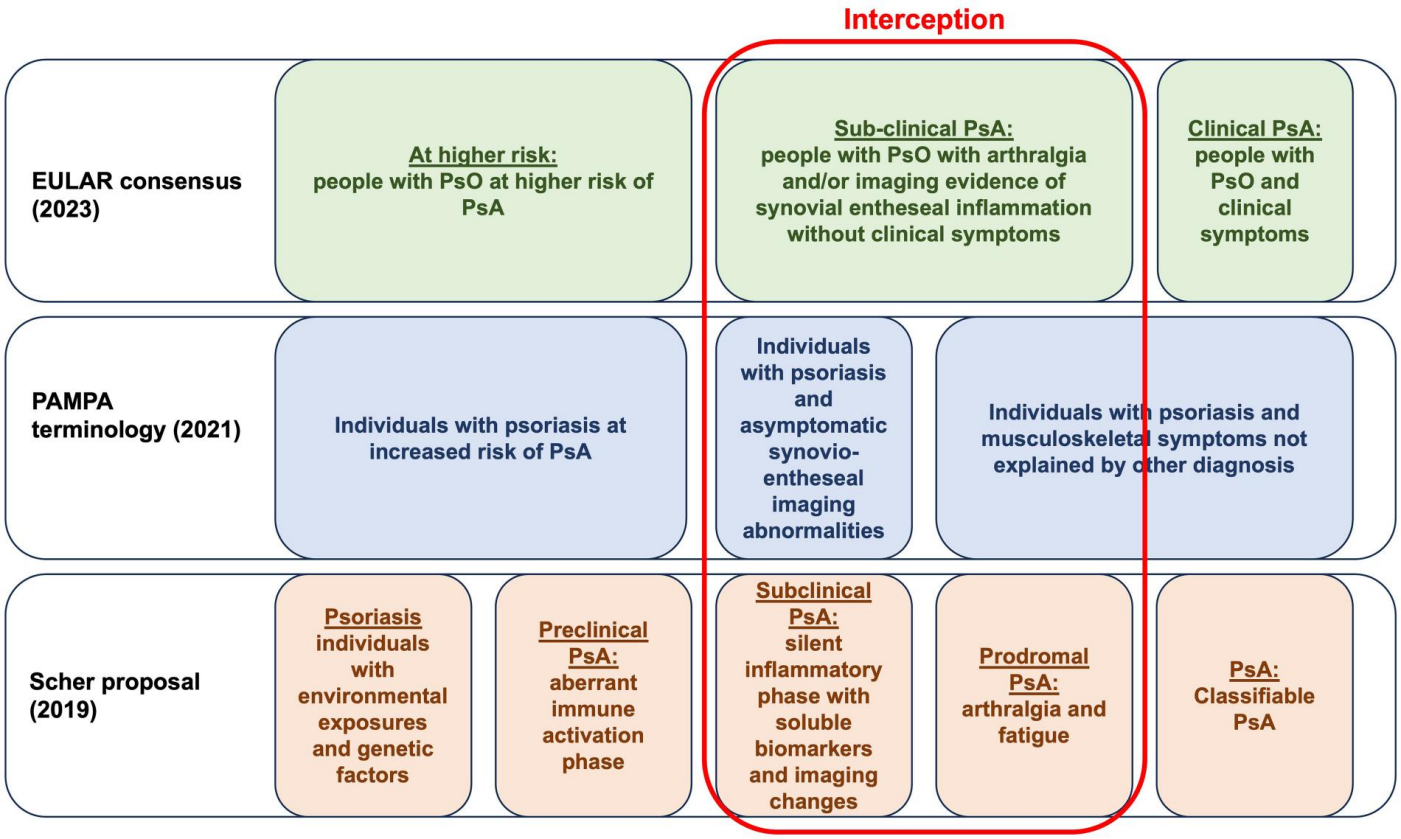
Phases proposed to explain the transition from PsO to PsA [21–23]. PAMPA: Preventing Arthritis in a Multicentre Psoriasis At-Risk Population; PsA: psoriatic arthritis; PsO: psoriasis

PsA usually appears in patients with skin PsO and is often preceded by a preclinical phase characterized by immunological abnormalities, arthralgia and imaging abnormalities before receiving a formal diagnosis [21]. Three working groups have proposed various terminologies to explain the transition from PsO to a formal diagnosis of PsA (Fig. 1). The first one, made by Scher *et al.* [21], proposed five distinct phases to explain the continuum PsO–PsA. The initial stage is represented by a patient with PsO and predisposing factors for PsA, such as genetics, obesity and PsO severity. However, there is no full consensus on these risk factors, which are represented in Table 1 and discussed below [21–23]. An intermediate phase is proposed only by Scher *et al.* (Fig. 1) and is characterized by the abnormal activation of the immune system, notably involving the IL-23–IL-17 axis and TNF production. This activation could be triggered by factors originating from cutaneous tissue, intestinal mucosa (specifically the microbiome) and/or the entheses [24]. Phase 3 corresponds with ‘subclinical PsA’ characterized by clinically asymptomatic imaging changes and phase 4 corresponds to a prodromal phase typified by arthralgia and fatigue. The final phase 5 is represented by a clinical diagnosis of PsA.

**Table 1.** Risk factors for PsA development according to different expert groups

	Scher *et al*. (2019) [21]	PAMPA terminology (2021) [22]	EULAR consensus (2023) [23]
Genetic factors	Yes	Yes	No
Family history	Yes	Yes	Yes
Obesity	Yes	Yes	Yes
Mechanical stress	Yes	No	Considered
Infections	Yes	No	Considered
Nail involvement	Yes	Yes	Yes
Psoriasis severity	Yes	Yes	Yes

Yes: recognized link; No: no recognized link; Considered: proposed as risk factor but not included in the main definition.

Then, in 2021, many of the same authors in the Psoriasis and Psoriatic Arthritis Clinics Multicentre Advancement Network (PPACMAN) and the Preventing Arthritis in a Multicentre Psoriasis At-Risk Population (PAMPA) study group proposed updated terminology (Fig. 1) [22]. The PAMPA proposal put less emphasis on genetics and immunological aberrations prior to PsA development since these are thus far poorly defined. This second iteration placed an emphasis on ‘synovio-entheseal complex inflammation’ but did not reference or define what this specifically meant, but we assume it references the functionally integration of the synovium and enthesis that leads to entheseal inflammation manifesting as synovitis [25]. The term ‘synovio-entheseal inflammation’ has been chosen rather than ‘enthesio-sinovial inflammation’ since the manifestation of joint swelling or synovitis is what is readily recognized [14]. It is noteworthy that imaging evidence of inflammation is present in up to 50% of PsO patients at any given time, yet the lifetime prevalence of PsA is 30%, which clearly attests to a large burden of potential inflammatory changes that will not develop PsA. Also, this group lacked a working definition for what constituted early PsA.

Recently in 2023, a EULAR task force proposed points to consider for the definition of clinical and imaging features suspicious for progression to PsA and developed a simplified nomenclature for the stages before PsA onset to be used in clinical trials aimed at PsA prevention (Fig. 1) [23]. The EULAR definition recognized that patients with PsO might be at potential risk of PsA development at some point, which is pragmatic and in keeping with clinical practice where new onset PsA may present with minimal or hitherto undiagnosed psoriasis. An important distinction in the EULAR definition was that some risk factors such as psoriasis, obesity, nail disease and family history were not imminent risk factors for PsA development but represented more long-term factors for planning prevention studies [23].

According to the EULAR task force, the second phase is represented by subclinical inflammation, which was named ‘subclinical PsA’ [23]. The subclinical PsA phase was defined as PsA with arthralgia on the basis that this is associated with a more imminent or immediate risk of PsA development with supportive data coming from the EULAR SLR and other papers [14, 26]. The EULAR taskforce also stated that imaging evidence of synovial or entheseal inflammation without clinical synovitis was part of the subclinical phase [23, 27]. In practice, many but not all patients with arthralgia have imaging abnormalities [14]. However, some limited data have suggested progression to PsA subjects with imaging changes alone [28, 29]. Hence EULAR used terminology that reflected this lack of certainty of the role of imaging by using the terminology that subclinical PsA represented arthralgia ‘and/or’ imaging evidence of inflammation [14, 23]. The EULAR taskforce felt that such simplification would help move the trial landscape in a positive way given that this arthralgia and imaging abnormal group would represent a best strategy for rationally designed studies including regression of arthralgia and improvement in imaging as an outcome or for the development of PsA that the EULAR taskforce also defined. Also, that abnormal ultrasound or MRI imaging is present in many ACPA^+^ arthralgia patients destined to develop RA supports similar potential mechanisms for synovitis development in PsA.

The presence of definite clinical inflammation represents an established disease. However, differences in the three proposals exist: Scher *et al.* consider clinical inflammation as the appearance of synovitis, enthesitis or dactylitis (Fig. 1). The PAMPA consensus combined the third (prodromal) and fourth phase (clinical inflammation) in only one stage defined as individuals with PsO and musculoskeletal symptoms not explained by other diagnosis. However, the EULAR taskforce only considered the presence of clinical synovitis for a PsA diagnosis. This was based on the EULAR systematic literature review [26] and data from 300 PsA arthralgia patients most of whom presented with a clinical synovitis as the presenting diagnosis of PsA [14]. So, for the first time EULAR suggested that the outcome of synovitis was the most likely presentation of PsA in arthralgia subjects with synovitis encompassing the dactylitic lesion and synovitis also representing an easier pathology to more objectively identify compared with enthesitis and axial inflammation.

## Identification of patients ‘at risk’ of PsA

Several risk factors of PsA in patients with PsO have been identified, though there is no final consensus (Table 1) [21–23]. Of note, initial signs or symptoms of joint inflammation such as arthralgia or subclinical imaging changes are not included in the table, since these elements may be considered more the first elements of PsA than risk factors *per se*.

Experts agree that individuals with PsO who have first-degree relatives with PsA have an increased risk of developing arthritis [21–23]. This genetic predisposition may be associated with MHC class-I alleles. Some genes have been identified, such as HLA-B*08, HLA-B*27, HLA-B*38 and HLA-B*39 [30]. However, the EULAR task force does not include genetics as a risk factor due to the incomplete understanding of the immunogenetic link to disease evolution (Table 1).

The presence of a psoriatic plaque is one of the most important clinical markers for future synovio-entheseal inflammation. Specific clinical features of PsO, such as the presence of nail disease, extent of PsO or its location (i.e. nail, scalp, or skin folds), can help identify patients with PsO ‘at risk’ of developing PsA [21, 31]. There is a notable association between obesity and the development of PsA, with obesity serving as a recognized independent risk factor for PsA [29]. The impact of obesity on PsA appears to be dose-dependent, with BMI associated with an increased risk of PsA development [26], possibly due to the increment in the biomechanical stress of enthesis.

Physical trauma is a well-known potential trigger of PsA. The Koebner phenomenon can occur in patients with PsO where psoriatic plaques emerge in regions exposed to trauma or microtrauma. Notably, enthesis shows microanatomical similarities with skin, including an avascular zone (fibrocartilage), making it susceptible to the Koebnerization responses [32].

At this stage of the PsO–PsA continuum, infections and other environmental factors (such as smoking and trauma) may also serve as additional causal factors in the development of PsA; however, there is currently no consensus on this point and the role of smoking is contentious as a PsA risk factor [21–23]. As stated, what differentiated the EULAR taskforce findings was the recognition that some risk factors are linked to PsA but not imminent or immediate risk whereas the presence of arthralgia may increase risk for formal PsA diagnosis.

## Physiopathology of the transition from PsO to PsA

**Figure 2.**
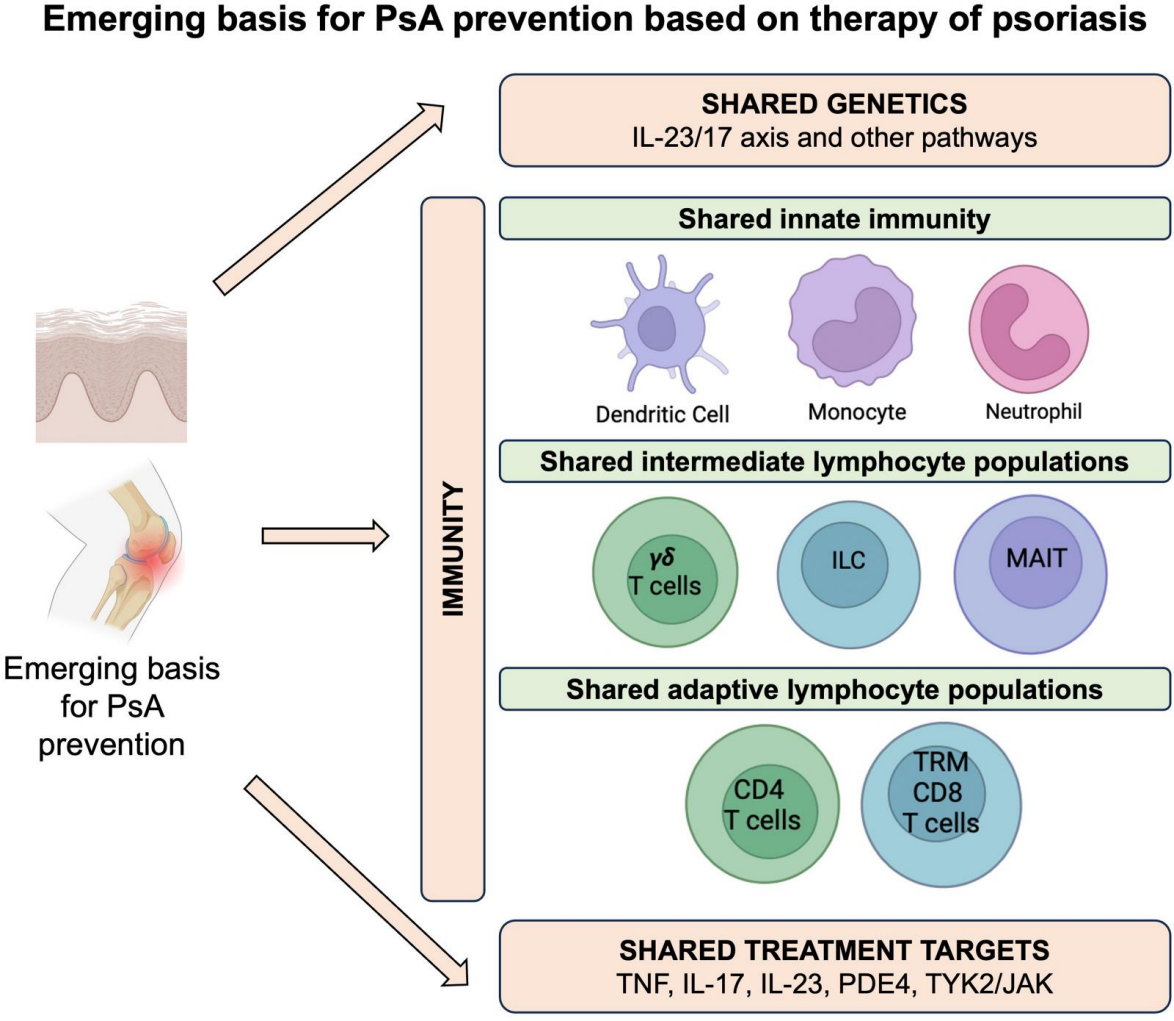
Emerging basis for PsA prevention based on therapy of psoriasis. ILC: innate lymphoid cells; JAK: Janus kinase; MAIT: mucosal-associated invariant T cell; PDE4: phosphodiesterase-4; PsA: psoriatic arthritis; TRM: tissue-resident memory T cell; TYK2: tyrosine kinase 2

Understanding the cellular and molecular pathways involved in the transition from PsO to synovio-entheseal inflammation presents an opportunity to establish the basis for preventing PsA in patients with PsO. It assumes that cutaneous immunity and entheseal immunity in subjects with psoriasis are very closely related or strongly overlapping and there is good supporting evidence for this (Fig. 2). However, the intestines and skin have large resident microbiotal communities but the entheses and joints are sterile points towards potentially disparate immune homeostasis between the enthesis and the skin. There are limited data on entheseal immunity in early PsA thus far, but data have started to emerge especially comparing immunity in the normal skin and normal enthesis.

In the pathophysiology of PsA, both the innate and adaptative immune systems play crucial roles. Like the skin, recent studies have demonstrated resident macrophages, neutrophils and plasmacytoid dendritic cells in the normal enthesis confirming innate immune populations at both locations. Innate immune or intermediate lymphocytes including group 3 innate lymphoid cells, γδ T cells and mucosal-associated invariant T cells are present in the entheses, intestinal mucosa and skin, pointing to similar innate effector mechanisms at all these sites [33–39]. These different innate lymphocytes can be activated by IL-23 via IL-23 receptors (IL-23R) present in these cells with induction of IL-17A, TNF and other cytokines, leading to an inflammatory process that, in the case of PsA, will predominate at the entheseal level [40]. The cytokine IL-17A (as well as IL-17F) is a pleiotropic effector cytokine, promoting intestinal homeostasis, joint and skin inflammation, bone destruction, and pathological bone formation (Fig. 2) [40, 41].

With respect to adaptive immunity, both conventional CD4 and CD8 T cells have been described in the normal enthesis [41]. That T cells, particularly CD8^+^ T, play a pivotal role in PsO pathogenesis is supported by the strong disease associations between HLA class I alleles and the expansion of oligoclonal CD8^+^ T cell populations and CD8^+^ T cell expansion has also recently been reported in PsA [40]. These conventional T- cells have considerable potential for elaboration of IL-17. It is now appreciated that conventional T cells (i.e. CD4 and CD8 T cells) may often represent tissue resident memory in both the joints and the skin [42, 43]. These shared immunological findings suggest that certain therapies may be effective in the phases preceding clinical PsA and/or the earliest stages of PsO-associated inflammatory arthritis [32].

## Interception of PsA

Currently, several biologic DMARDs (bDMARDs) blocking specific cytokines (e.g. TNF, IL-17, IL-23) are licensed for use in either PsO or PsA (Table 2) [44]. The concept of interception refers to the prevention of clinical PsA in patients with PsO by treating patients in the third and fourth stages of the transition PsO–PsA (i.e. those asymptomatic with imaging abnormalities or with a prodromal PsA) (Fig. 1). However, in patients with PsO but without joint symptoms, imaging abnormalities are very common. Thus, using imaging techniques in all patients with PsO is not recommended and should only be performed in PsO patients with arthralgia. There are now data indicating that treating PsO patients with targeted drugs could have the potential to intercept the development of PsA [45] (Table 3).

**Table 2.** Drugs approved and licenced for use either in PsO or PsA in 2024 [44]

Inhibition	Drug	Approved in PsO	Approved in PsA
TNF	Infliximab	Yes	Yes
	Adalimumab	Yes	Yes
	Golimumab	No	Yes
	Certolizumab	Yes	Yes
	Etanercept	Yes	Yes
IL-17A	Secukinumab	Yes	Yes
	Ixekizumab	Yes	Yes
	Brodalumab	Yes	No
IL-17A/F	Bimekizumab	Yes	Yes
CTLA-4	Abatacept	No	Yes
IL-12/23	Ustekinumab	Yes	Yes
IL-23	Guselkumab	Yes	Yes
	Risankizumab	Yes	Yes
	Tildrakizumab	Yes	No
PDE4	Apremilast	Yes	Yes
JAK	Tofacitinib	No	Yes
	Upadacitinib	No	Yes

No: drug not currently approved/licensed; Yes: drug currently approved/licensed. JAK: Janus kinases; PDE4: phosphodiesterase 4; PsA: psoriatic arthritis; PsO: psoriasis.

**Table 3.** Interception of PsA: studies ordered by increasing number of patients

**Study**	Drug used and comparator	*n* patients in treatment and comparator arms	Incidence of PsA per 100 patient-years in the treatment group	Incidence of PsA per 100 patient-years in the comparator group	Risk of PsA in treatment *vs* comparator
Gisondi *et al.* (2022) [46]	bDMARDs *vs* phototherapy	234 *vs* 230	1.20 (95% CI: 0.77, 1.89)	2.17 (95% CI: 1.53, 3.06)	HR 0.53 (95% CI: 0.30, 0.94)
Acosta-Felquer *et al.* (2022) [47]	bDMARDs *vs* csDMARDs *vs* topics	103 *vs* 229 *vs* 1719	0.43 (95% CI: 0.11, 1.70)	csDMARDs: 1.20 (95% CI: 0.56, 2.80)	bDMARDs *vs* csDMARDs: IRR 0.35 (95% CI: 0.04, 1.96)
				Topics: 1.67 (95% CI: 1.50, 1.90)	bDMARDs *vs* topics: IRR 0.26 (95% CI: 0.03, 0.94)
Rosenthal *et al.* (2022) [48]	bDMARDs *vs* no bDMARDs	663 *vs* 663	—	—	HR 0.72 (95% CI: 0.53-0.97)
Singla *et al.* (2023) [49]	TNFi *vs* IL-12/23i *vs* IL-23i *vs* IL-17i	10037 *vs* 2914 *vs* 1149 *vs* 1401	TNFi: 3.83	IL-12/23i: 2.21	IL-12/23i *vs* TNFi: HR 0.58 (95% CI: 0.43, 0.76)
				IL-23i: 2.16	IL-23i *vs* TNFi: HR 0.41 (95% CI: 0.17, 0.95)
				IL-17i: 3.20	IL-17i *vs* TNFi: HR 0.86 (95% CI: 0.54, 1.38)
Lebwohl *et al.* (2023) [50]	IL-23i *vs* IL-17i *vs* IL-12/23i *vs* TNFi	2330 *vs* 819 *vs* 1100 *vs* 2895	—	—	IL-23i *vs* IL-17: HR 0.51 (95% CI: 0.29, 0.87)
					IL-23i *vs* IL-12/23: HR 0.55 (95% CI: 0.32, 0.92)
					IL-23i *vs* TNFi: HR 0.44 (95% CI: 0.29, 0.67)
Meer *et al.* (2022) [51]	Biologic therapy *vs* oral systemic therapy or phototherapy	14569 *vs* 20321	7.73	Oral systemic: 6.20	Biologic therapy *vs* oral systemic therapy or phototherapy: HR 4.48 (95% CI: 4.23, 4.75)
				Phototherapy: 2.61	

A HR below 1 indicates a ‘protective’ effect of the drug on the incidence of PsA. bDMARD: biologic DMARD; csDMARD: conventional synthetic DMARD; HR: hazard ratio; IL-17i: IL-17 inhibitor; IL-23i: IL-23 inhibitor; IRR: incidence risk ratio; PsA: psoriatic arthritis; TNFi: TNF inhibitor.

### Initial smaller studies

In a study by Gisondi *et al.* that involved 464 patients with PsO, the annual incidence rate of PsA was found to be lower in patients treated with bDMARDs compared with patients receiving phototherapy [46], confirming a potential delay or reduction in the risk of incident PsA in patients with moderate-to-severe chronic plaque PsO (Table 3) [46]. Similarly, Acosta-Felquer *et al.* found in 1719 PsO patients that the risk of developing PsA in those treated with bDMARDs was significantly lower compared with topical treatments, but not significantly different from those treated with conventional synthetic DNARDs [47]. This is very interesting given that conventional DMARDs are not thought to be effective for enthesitis but probably prevent its evolution, which raises novel questions about early PsA therapy. Finally, Rosenthal *et al.* found similar results in 1326 patients within a 10-year follow-up period [48].

### Recent larger studies

Singla *et al.* analysed 15 501 patients with PsO from a national sample in the USA, derived from the electronic health records of the TriNetX database (Cambridge, MA, USA) [49]. Among these patients, 976 (6.3%) developed inflammatory arthritis over a mean follow-up of 2.4 years. In multivariable regression analyses, the risk of developing inflammatory arthritis was significantly lower in patients prescribed bDMARDs not targeting TNF, specifically IL-12/23 inhibitors or IL-23 compared with those prescribed TNF inhibitors. Thus, treatment with IL-12/23 inhibitors or IL-23 inhibitors was associated with a reduced risk of progression to inflammatory arthritis compared with TNF inhibitors and IL-17 inhibitors. However, we must consider that these drugs are also effective in treating PsA. Thus, patients may have developed PsA, but they don’t show symptoms because the arthritis is also being treated. Future studies to evaluate whether the PsA appears after withdrawing these drugs would be useful to confirm this hypothesis. A recent abstract suggested a difference among the modes of action. Lebwohl *et al.* analysed 7144 biologic-naïve PsO patients and found that patients treated with IL-23 inhibitors were significantly less likely to develop PsA in comparison with patients treated with IL-17, IL-12/23 or TNF inhibitors [50]. This work is not yet published as full text. In contrast, Meer *et al.* analysed 193 709 patients with PsO from a US claims registry and found an increased incidence of PsA among users of biologics compared with those initiating oral DMARDs/phototherapy [51]. These findings are contradictory compared with the conclusions from previous studies, which raises some questions (Table 3).

Of note, all of these results are from claims databases or retrospective studies. There is a need for caution when interpreting outcomes from retrospective studies, since several confounders and sources of bias should be taken into consideration, such as confounding by indication and the protopathic bias (when a drug is prescribed for an early manifestation of the disease that has not yet been diagnostically confirmed) [52]. Ongoing randomized placebo-controlled, interventional, preventive trials will provide stronger evidence on the role of bDMARDs in preventing clinical musculoskeletal inflammation [53].

## Conclusion

The current focus of many international efforts is on pre-PsA, in the context of some promising findings is PsA disease interception. Recent definitions of ‘pre-PsA’ aim to facilitate research focused on the various stages preceding clinical PsA and its interception. We believe that the EULAR strategy with a subclinical PsA phase, the middle of three stages, offers a robust method for studies looking into PsA prevention. The next years will show if a homogenized use of nomenclature facilitates comparisons. In terms of intercepting PsA and where the knowledge is at, two large recent analyses evidenced conflicting results whereas smaller studies were mostly positive. More knowledge on whether PsA can be intercepted by targeting specific pathways involved in the pathogenesis of the disease is needed.

## Data Availability

There are no new data associated with this article.
